# Can the digestibility of corn distillers dried grains with solubles fed to pigs at two stages of growth be enhanced through management of particle size using a hammermill or a roller mill?

**DOI:** 10.1093/tas/txaa171

**Published:** 2020-09-12

**Authors:** Jesus A Acosta, Amy L Petry, Stacie A Gould, Cassandra K Jones, Charles R Stark, Adam C Fahrenholz, John F Patience

**Affiliations:** 1 Department of Animal Science, Iowa State University, Ames, IA; 2 Department of Animal Sciences and Industry, Kansas State University, Manhattan, KS; 3 Prestage Department of Poultry Science, North Carolina State University, Raleigh, NC

**Keywords:** feed processing, feeding grinding, fiber digestibility, particle size distribution, swine

## Abstract

The objective of this study was to determine the impact of reducing the mean particle size (PS) of corn distillers dried grains with solubles (DDGS) with a hammermill (HM) or with a roller mill (RM) on the apparent total tract digestibility (ATTD) of dry matter (DM), gross energy (GE), N, acid hydrolyzed ether extract (AEE), and fiber components in growing and finishing pigs. Twenty-four growing barrows were housed in individual pens and were randomly assigned to a 3 × 2 factorial design (*n* = 8): three grinding methods [either corn DDGS ground with an HM to a PS of 450 μm; corn DDGS ground with an RM to a PS of 450 μm; and corn DDGS with a PS of 670 μm (not further ground)] and two body weight (BW) periods (growing pigs with an average initial BW of 54.7 ± 0.9 kg, and finishing pigs with an average initial BW of 107.8 ± 1.5 kg BW). Fecal samples were collected for each BW period in the last 3 d of an 11-d feeding period. Titanium dioxide was used as an indigestible marker. Digestibility data were analyzed using the MIXED procedure of SAS. Results showed that finishing pigs tended to have better ATTD of DM than growing pigs (*P* = 0.09) and had increased ATTD of GE and N than growing pigs (*P* = 0.03 and *P* < 0.01, respectively). On the other hand, growing pigs had better ATTD of AEE than finishing pigs (*P* = 0.01). Pig BW period did not affect the ATTD of neutral detergent fiber (NDF), acid detergent fiber (ADF), and hemicellulose. Reducing the mean PS of corn DDGS with either HM or RM (from 670 to 450 µm) improved the ATTD of DM and GE (*P* < 0.01 and *P* < 0.01), tended to improve the ATTD of N (*P* = 0.08), and improved the ATTD of AEE (*P* < 0.01). No effect of reducing PS was observed for the ATTD of NDF, ADF, or hemicellulose. There were no differences between HM and RM in any of the ATTD variables tested. In conclusion, reducing PS of corn DDGS from 670 to 450 μm either with an HM or with an RM improved the digestibility of DM, GE, and AEE and modestly improved the digestibility of N in growing and finishing pigs. However, reducing the PS of corn DDGS did not affect the digestibility of fiber components.

## INTRODUCTION

Corn distillers dried grains with solubles (DDGS) is a coproduct of the production of ethanol through a dry grind process (Belyea et al., 2010). As an ingredient, corn DDGS is characterized as a good source of crude protein (~28%), digestible phosphorus (~0.40%), ether extract (depending on processing; 3.5–12%), and definitively an abundant source of insoluble fiber [~32%, neutral detergent fiber (NDF); NRC, 2012; Gutierrez et al., 2016]. Unlike grains such as corn and wheat, corn DDGS is present in ground form because grinding is the initial step in ethanol production [usually using a hammermill (HM); Rausch et al., 2005; Wongsagonsup and Jane, 2017]. However, the mean particle size (PS) is highly variable among sources (~450–900 μm; Liu, 2008, 2009a).

Regrinding corn DDGS may represent an opportunity to enhance its feeding value for pigs, especially for larger PS (approximately from 600 to 900 μm). The rationale is to improve feeding value by decreasing PS, thus increasing access to digestive enzymes and the digestibility of nutrients (Giesemann 1990; Wondra et al., 1995). Another benefit could be greater homogeneity of the diet (Ramirez et al., 2012). Liu et al. (2012) reported the benefit of grinding corn DDGS (from 818 to 308 μm) in terms of digestibility of dry matter (DM) and gross energy (GE). However, more research is needed not only to support this evidence but also to assess the effect of the PS reduction on fiber digestibility.

Additionally, two other major factors need to be studied. First, it is necessary to clarify if grinding methods (GMs), such as HM or roller mill (RM), are equally effective. The digestibility of corn and wheat has been reported to differ by using an HM compared with an RM (Acosta et al., 2020a, 2020b). Second, digestion improves as animals grow (Noblet and Henry, 1993; Acosta et al., 2020b). This has been attributed to a more developed gastrointestinal tract with greater digestive capacity. With this in mind, it begs the question if the response to PS reduction and to GM varies at different growth stages.

Therefore, the objective of this study was to determine if reducing PS of corn DDGS from 670 to 450 μm with an HM or an RM would improve the digestibility of energy and nutrients in both growing and finishing pigs. We hypothesized that reducing mean PS either with an HM or an RM would result in increased digestibility of energy and nutrients for both growing and finishing pigs but with greater enhancement using the former.

## MATERIALS AND METHODS

Experimental procedures were approved by the Institutional Animal Care and Use Committee at Iowa State University (2-14-7731-S) and adhered to the Guide for the Care and Use of Agricultural Animals in Research and Teaching (FASS, 2010).

### Animals Housing and Experimental Design

This experiment was conducted at the Iowa State Swine Nutrition Farm (Iowa State University, Ames, IA). A total of 24 barrows, the progeny of C22 or C29 sows × 337 terminal sires (PIC Inc., Hendersonville, TN), were randomly assigned to a 3 × 2 factorial design. The first factor involved three GM/PS categories, namely corn DDGS that was not further ground and having a PS of 670 μm, corn DDGS ground to a PS of 450 μm using an HM, and corn DDGS ground to a PS of 450 μm using an RM. The second factor was body weight (BW) periods: growing pigs with an average initial BW of 54.7 ± 0.9 kg and finishing pigs with an average initial BW of 107.8 ± 1.5 kg BW. There were eight observations per treatment.

Pigs were housed in an environmentally controlled room with individual pens, each including a partially slatted concrete floor, an automatic dry self-feeder, and a cup drinker. The daily feed allowance provided 2.5 (for the two lightest blocks on each treatment) or 2.7 (for the remaining six pigs) times the estimated daily maintenance energy requirement (NRC, 2012) for each growth stage. Feed was offered in mash form twice daily at 0800 and 1600 hours in equal-sized quantities for 11 d. All pigs had ad libitum access to water.

The same 24 animals remained on test over the two BW categories, but they were rerandomized to PS category following the conclusion of the growing period. Pigs were not permitted to receive the same experimental diet in the growing and in the finishing periods. For the 45 d between the two BW periods, pigs were fed a typical commercial growing diet, based on corn, soybean meal, and corn DDGS, which met or exceeded the nutrient requirements as defined by the NRC (2012).

### Experimental Diets

The experimental diets were manufactured at the O.H. Kruse Feed Technology Innovation Center (Kansas State University, Manhattan, KS). A commercial source of corn DDGS [8.4% acid hydrolyzed ether extract (AEE) and 29.7% crude protein (CP)] was ground to a PS of 450 µm either with an HM (model 22115, Bliss Industries, Ponca City, OK) or with an RM (model 924; RMS Roller Grinder, Harrisburg, SD) or it was used as it arrived with no further processing (PS of 670 µm).

The experimental diets contained 45% of the three samples of corn DDGS and 51% of corn ground to 500 µm using a RM. Titanium dioxide (TiO2) was included in the diet as an indigestible marker (Table 1). Diets were formulated to ensure that there were no other plant- or animal-based proteins, fats, or synthetic sources of amino acids. This approach ensured that corn origin ingredients were the only source of amino acids, carbohydrates, and energy in the diets and the difference between the diets was achieved through the three GM/PS combinations.

**Table 1.** Ingredient composition of the experimental diets^*a*^

Ingredient	Amount, %
**Corn** ^^*b*^^	**51.91**
**Corn DDGS**	**45.00**
**Monocalcium phosphate**	**0.35**
**Calcium carbonate**	**1.44**
**Salt**	**0.50**
**Vitamin premix** ^^*c*^^	**0.20**
**Mineral premix** ^*^d^*^	**0.20**
**Titanium dioxide**	**0.40**

^*a*^The experimental diets corresponded to three PS categories, corn DDGS ground at 450 µm with a hammer mill, corn DDGS ground at 450 µm with an RM, and corn DGGS not further ground with a mean PS of 670 µm.

^*b*^Corn ground with an RM to a PS of 500 µm.

^*c*^Vitamin premix provided the following (per kilogram diet): 6,125 IU of vitamin A; 700 IU of vitamin D3; 50 IU of vitamin E; 3 mg of menadione (to provide vitamin K); 11 mg of riboflavin; 27 mg of d-pantothenic acid; 0.05 mg of vitamin B12; and 56 mg of niacin.

^*d*^Mineral premix provided the following (per kilogram diet): 220 mg of Fe (ferrous sulfate); 220 mg of Zn (zinc sulfate); 52 mg of Mn (manganese sulfate); 22 mg of Cu (cooper sulfate); 0.4 mg/kg of I (calcium iodate); and 0.4 mg/kg of Se (sodium selenite).

### Sample Collection, Chemical Analyses, and Calculations

The PS size distribution of each corn DDGS grinding category was measured according to the methods of Kalivoda et al. (2017) at the Kansas State University Swine Nutrition Laboratory (Manhattan, KS). Briefly, a 100 ± 5 g corn DDGS subsample was obtained by using a riffle divider and an analytical scale. Each subsample was shaken with 0.5 g of dispersion agent for 15 min using a sieve shaker (model Ro-Tap RX−26, W. S. Tyler Industrial Group, Mentor, OH) furnished with 13 sieves (U.S. standard sieve nos. 6, 8, 12, 16, 20, 30, 40, 50, 70, 100, 140, 200, and 270) and a pan equipped with sieve agitators (model SSA-58, Gilson Company Inc., Lewis Center, OH). Material retained in the middle sieve fractions (sieve nos. 20, 30, 40, 50, and 70) were harvested for further laboratory analyses. The geometric mean diameter and geometric SD were calculated using the ANSI/ASAE S319.2 (American Society of Agricultural and Biological Engineers [ASABE], 1995) standard method.

At the time of mixing, 10 diet subsamples were collected at the feed mill and then thoroughly homogenized and subsampled. Fresh fecal subsamples were obtained twice daily via grab sampling from the floor of the pen at 0930 and 1630 hours during d 9–11 of each test period, placed in prelabeled plastic bags, and immediately frozen at −20 °C. Once the collection was completed, fecal samples were homogenized, dried to a constant weight in an oven at 65 °C (Jacobs et al., 2011), and ground in a Wiley mill through a 1-mm screen (Model ED-5, Thomas Scientific Inc., Swedesboro, NJ). Feed and sieve fractions were divided into two subsamples, one ground through a 1-mm screen and the second through a 0.5-mm screen using a centrifugal mill (Model ZM1, Retsch Inc., Newton, PA). All fecal, feed, and sieve fraction samples were kept in plastic bags in desiccator cabinets to maintain constant moisture content until all chemical assays were completed.

Images depicting the topography and PS of the experimental diets were captured using field emission scanning electron microscopy (Roy J. Carver High-Resolution Microscopy Facility, Iowa State University, Ames, IA) following the procedures previously described by Acosta et al. (2020a, 2020b).

Samples of sieve fractions, feed, and feces were analyzed at the Monogastric Nutrition Laboratory (Iowa State University, Ames, IA). Assays included DM using a drying oven (method 930.15; AOAC, 2007) and N using the combustion method (Nitrogen Determinator; model TruMac N, Leco Corporation, St. Joseph, MI; method 990.03; AOAC, 2007). The standard for calibration was ethylenediaminetetraacetic acid (9.57% N; Leco Corporation, St. Joseph, MI) and determined to contain 9.58 ± 0.02% N. Crude protein was calculated as N × 6.25. AEE was determined using a SoxCap hydrolyzer (model SC 247) and a Soxtec fat extractor (model 255), Foss, Eden Prairie, MN (method 968; AOAC, 2007). Starch content was determined using the 0.5-mm ground samples using the Megazyme total starch assay kit (Wicklow, Ireland; modified method 996.11; AOAC 1996). Acid detergent fiber (ADF) and NDF content of feed and fecal samples were determined using an Ankom automated fiber analyzer (model 2000, Macedon, NY) according to a modified method from Van Soest and Robertson (1980). Hemicellulose content was determined by subtracting ADF from NDF. Gross energy was determined using an isoperibolic bomb calorimeter (Model 6200, Parr Instrument Co., Moline, IL). Benzoic acid (6,318 kcal GE/kg; Parr Instruments, Moline, IL) was used as the standard for calibration and was determined to contain 6,323 ± 1.8 kcal GE/kg. Titanium dioxide (only for feed and fecal samples) was determined colorimetrically using a spectrophotometer (Model Synergy 4, BioTek, Winooski, VT) according to the method of Leone (1973). The apparent total tract digestibility (ATTD) of DM, GE, CP, AEE, NDF, ADF, and hemicellulose was calculated using the following equation (Oresanya et al., 2008):

$$\begin{array}{l} \begin{array}{l} ATTD,\;\%=[100-[100\;\times \;\left(\frac{\%\;TiO2\;in\;feed}{\%\;TiO2\;in\;feces}\right)\\ \;\times \left(\frac{concentration\;of\;component\;in\;feces}{concentration\;of\;component\;in\;feed}\right)]] \end{array} \end{array}$$

### Statistical Analysis

Data were analyzed according to the mixed model:

$$\begin{array}{l} y_{ijk}=\mu +\tau_{i}+\lambda_{j}+\theta_{k}+{\left(\tau \lambda \right)}_{ij}+\delta_{k}+\epsilon_{ijk} \end{array}$$

where y_ijk_ represents the observed value for the lth experimental unit within the ith level of GM category and jth level of BW period of the lth pig; μ is the overall mean; τ represents the fixed effect of GM category (*i* = 1–3); λ represents the fixed effect of BW period (*k* = 1, 2); τλ represents the interaction effect between GM and BW period; δ represents the random effect of the lth pig (l = 1–8); $\epsilon_{ijk}$ is the associated variance as described by the model for$\;y_{ijk}$ assuming δ ~Ν (0, ***I ***$\sigma^{2}$) and; $\epsilon_{ijkl}$ ~Ν(0,$\;I\sigma^{2}$), where***I*** is the identity matrix.

The pig was the experimental unit for all analyses. The UNIVARIATE procedure of SAS (SAS Inst., Inc., Cary, NC) was used to verify normality, the homogeneity of residual variance from the reported model, and to identify statistical outliers (>3 SD from the mean). The model was analyzed using the MIXED procedure of SAS. The effects were considered statistically significant, with *P-*values ≤0.05 and trends with *P-*values between 0.05 and 0.10. The chemical composition of sieve fractions is presented as descriptive statistics, as there was no replication.

## RESULTS

### Physical Composition of Corn DDGS and Chemical Composition of Experimental Diets

**Figure 1.**
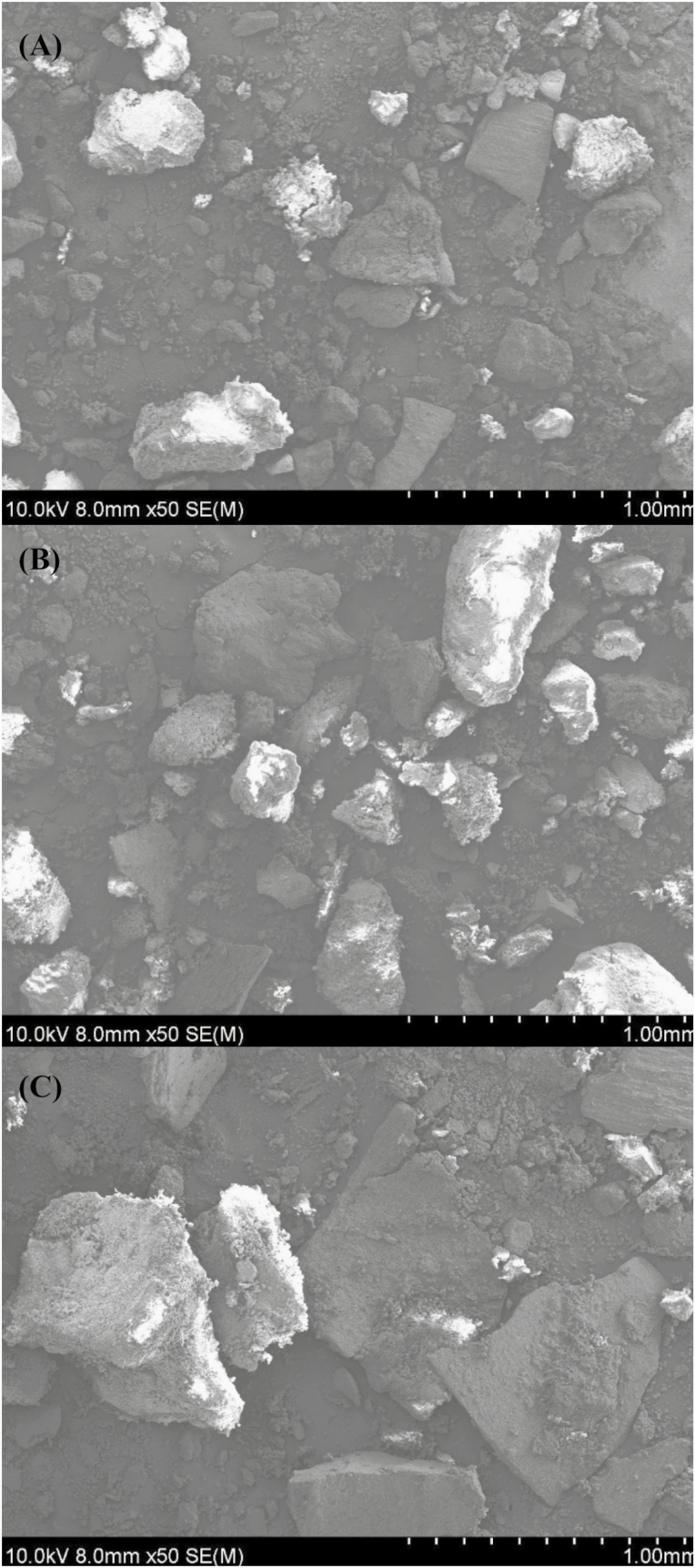
Field emission scanning electron microscopic images (×50) of particles from diets with 55% corn ground with an RM to a mean PS of 500 µm and 45% of corn DDGS ground to a mean PS of (A) 450 μm with an HM, (B) 450 μm with an RM, and (C) 670 μm used as arrived.

**Figure 2.**
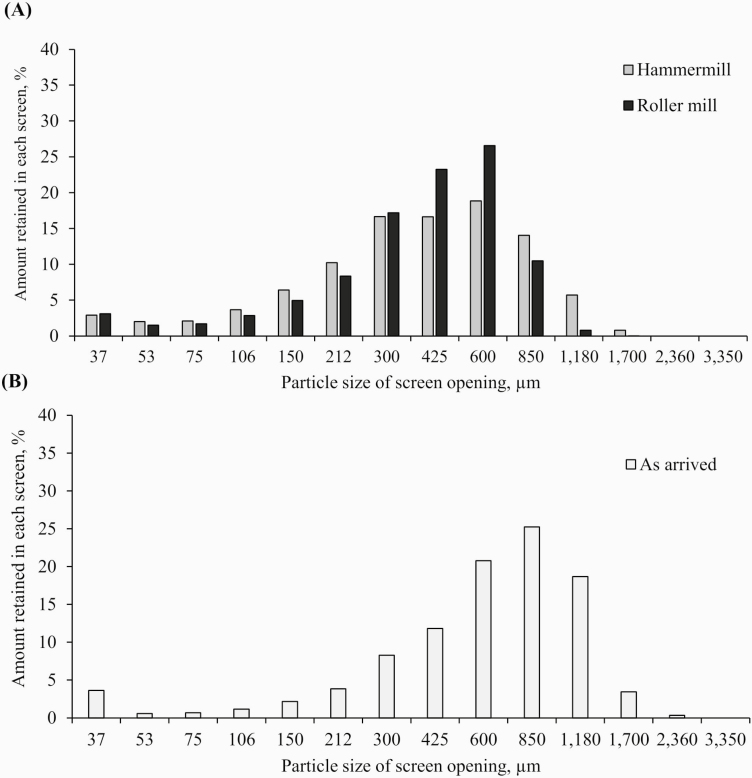
Particle size distribution of corn DDGS (A) ground at 450 µm (with an HM or with an RM), or (B) as arrived (no further ground) with a 670 µm mean PS (n = 1 for all samples).

Scanning electron microscopy images of diets containing DDGS ground to 450 μm with either an HM or an RM showed an evident decrease in the size of particles compared with the diet with no reground corn DDGS (670 μm of mean PS; Fig. 1). However, no distinguishable or evident differences in particle shape were observed between HM and RM. The PS distribution of corn DDGS ground with a HM had a similar percentage of fine particles but slightly larger particles than RM. On the other hand, RM had a higher percentage of particles within the mean PS range than the HM (Fig. 2).

The corn DDGS arrived with a mean PS of 670 μm. The mean PS of corn DDGS ground with either an HM or an RM were slightly lower than the targeted PS (450 μm) but very similar to each other (429 and 426 μm, respectively; Table 2). The determined SD of PS ranged from 2.1 to 2.3 among dietary treatments. As expected, the chemical composition of the experimental diets was similar across GMs (Table 3).

**Table 2.** Geometric mean diameter (d_gw_) and geometric SD (S_gw_) of corn DDGS ground with an HM, with an RM, or no further ground (NG)^a^

	Targeted PS, μm	450	450	^—^
Item		HM450	RM450	NG670
**Mean PS, µm**		**429**	**426**	**670**
**SD of PS**		**2.3**	**2.1**	**2.3**

^a^Variables were determined according to ANSI/ASAE S319.2 (American Society of Agricultural and Biological Engineers [ASABE], 1995) standard method for PS analysis at the Kansas State University Swine Nutrition Laboratory.

**Table 3.** Analyzed chemical composition of the experimental diets^*a*^, as-fed basis

Item	HM 450	RM 450	NG 670
DM, %	91.2	91.1	90.9
GE, Mcal/ kg	4.05	4.06	4.06
CP, %	16.5	16.6	17.4
AEE, %	5.0	5.2	5.2
Starch, %	40.9	41.1	39.1
ADF, %	5.4	5.4	5.6
NDF, %	14.2	14.1	15.1

^*a*^HM 450 = corn DDGS ground with an HM at 450 µm; RM 450 = corn DDGS ground with an RM at 450 µm; and NG 670 = corn DDGS with a 670 µm mean PS no further ground.

### ATTD of Dietary Components

**Figure 3.**
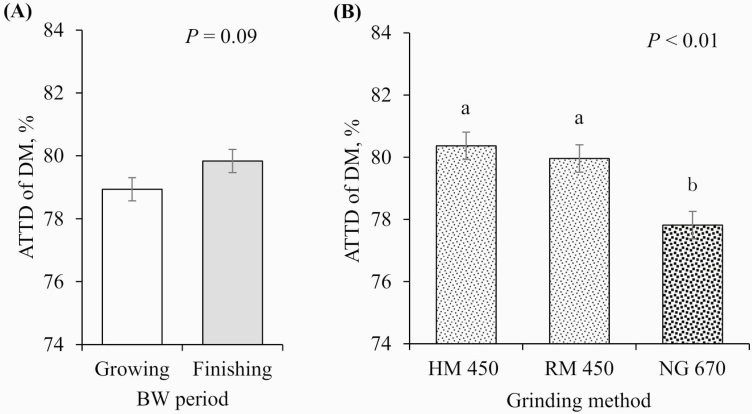
Effects of (A) BW period (growing pigs, average BW = 54 kg; finishing pigs, average BW = 106 kg) and (B) GM: HM 450 = corn DDGS ground with an HM at 450 µm, RM 450 = corn DDGS ground with an RM at 450 µm, and NG 670 = corn DDGS no further ground with a mean PS of 670 µm on the ATTD of DM. a–b: values with differing superscripts denote differences (*P* ≤ 0.05). There was no interaction between BW period and GM (*P* = 0.376).

**Figure 4.**
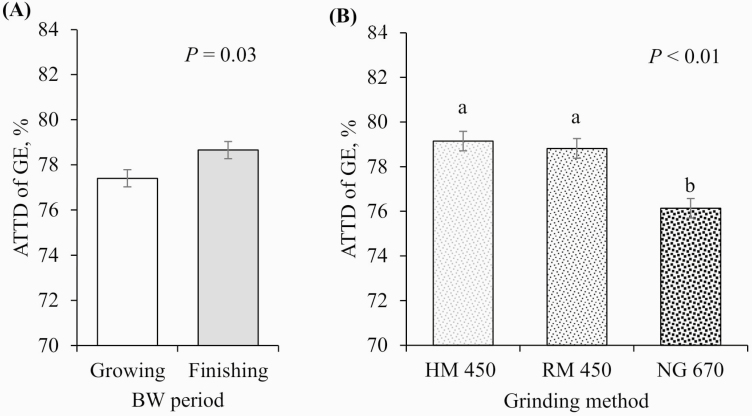
Effects of (A) BW period (growing pigs, average BW = 54 kg; finishing pigs, average BW = 106 kg) and (B) GM (HM 450 = corn DDGS ground with an HM at 450 µm, RM 450 = corn DDGS ground with an RM at 450 µm, and NG 670 = corn DDGS with a 670 µm mean PS no further ground) on the ATTD of GE. a–b: values with differing superscripts denote differences (*P* ≤ 0.05). There was no interaction between BW period and GM (*P* = 0.120).

**Figure 5.**
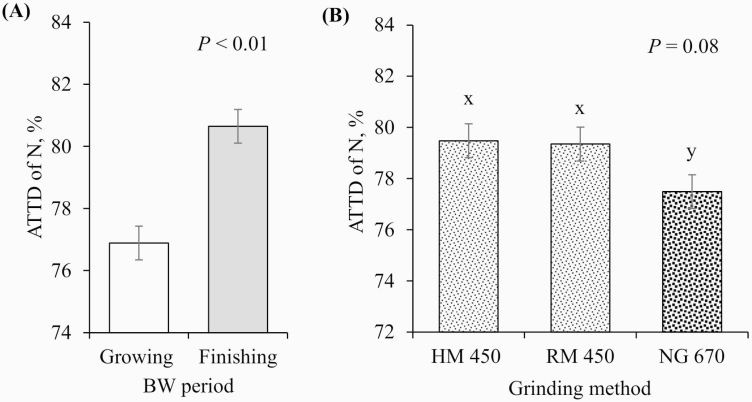
Effects of (A) BW period (growing pigs, average BW = 54 kg; finishing pigs, average BW = 106 kg) and (B) GM (HM 450 = corn DDGS ground with an HM at 450 µm, RM 450 = corn DDGS ground with an RM at 450 µm, and NG 670 = corn DDGS with a 670 µm mean PS no further ground) on the ATTD of N. x–y: values with differing superscripts denote differences (*P* ≤ 0.10). There was no interaction between BW period and GM (*P* = 0.419).

**Figure 6.**
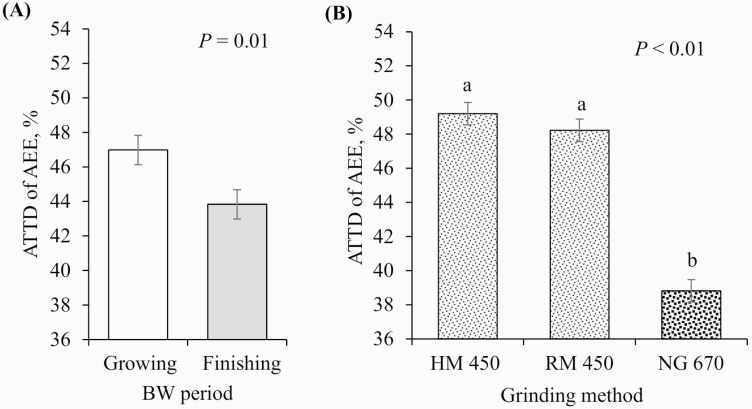
Effects of (A) BW period (growing pigs, average BW = 54 kg; finishing pigs, average BW = 106 kg) and (B) GM (HM 450 = corn DDGS ground with an HM at 450 µm, RM 450 = corn DDGS ground with an RM at 450 µm, and NG 670 = corn DDGS with a 670-µm mean PS no further ground) on the ATTD of AEE. a–b: values with differing superscripts denote differences (*P* ≤ 0.05). There was no interaction between BW period and GM (*P* = 0.272).

No interactions between pig BW period and GM were observed for any of the variables tested (*P* > 0.10); therefore, the main effects of the BW period and GM are presented. Finishing pigs tended to have better ATTD of DM than growing pigs (79.8% vs. 78.9%, respectively; *P* = 0.09; Fig. 3A) and had increased ATTD of GE (78.7% vs. 77.4%, respectively; *P* = 0.03; Fig. 4A) and N (80.7% vs. 76.9%, respectively; *P* < 0.01; Fig. 5A). In contrast, growing pigs had better ATTD of AEE than finishing pigs (46.9 vs. 43.8%, respectively; *P* = 0.01; Fig. 6A). Surprisingly, pig BW period did not affect the ATTD of NDF, ADF, or hemicellulose (Table 4).

**Table 4.** Effects of BW period and GM on the ATTD of fiber fractions^*a*^

	BW period	Growing			Finishing				*P*-value		
Item	GM	HM 450	RM 450	NG 670	HM 450	RM 450	NG 670	SEM	GM	BW Period	GM × period
NDF		46.6	44.9	47.0	50.2	48.5	44.7	1.4	0.477	0.335	0.727
ADF		58.7	57.1	58.0	61.8	60.6	56.5	1.8	0.306	0.267	0.374
Hemicellulose		39.3	37.3	40.4	43.2	40.9	37.6	3.4	0.763	0.576	0.565

^*a*^BW period was either growing pigs (average BW = 54 kg) or finishing pigs (average BW = 106 kg); GM: HM 450 = corn DDGS ground with an HM at 450 µm, RM 450 = corn DDGS ground with an RM at 450 µm, and NG 670 = corn DDGS no further ground with a mean PS of 670 µm.

On GM, there were no differences between the HM and the RM in any of the ATTD variables tested. However, reducing the mean PS of corn DDGS (from 670 to 450 µm) improved the ATTD of DM (77.8% vs. 80.4% and 80.0% for HM and RM, respectively; *P* < 0.01; Fig. 3B) and GE (76.1% vs. 79.2% and 78.8% for HM and RM, respectively; *P* < 0.01; Fig. 4B), tended to improve the ATTD of N (77.5% vs. 79.5% and 79.4% for HM and RM, respectively; *P* = 0.08; Fig. 5B), and improved the ATTD of AEE (38.8% vs. 49.2% and 48.2% for HM and RM, respectively; *P* < 0.01; Fig. 6B). No effect of reducing PS was observed for the ATTD of NDF, ADF, and hemicellulose.

### Chemical Composition of Sieve Fractions

Description of the chemical composition of the sieve fractions must be interpreted with care since these values had no replication; thus, they are presented as simple means (Table 5). Corn DDGS ground with an HM and RM showed similar concentrations of DM, GE, and starch across the various sieve sizes. However, the concentration of NDF appeared to be higher in the 212-µm sieve in the RM processed material; the values tended to be lower for all other screen sizes. Of course, there is no way of knowing if this difference is real or an artifact; further research is required to confirm this observation.

**Table 5.** Analyzed chemical composition of sieve fractions of corn DDGS ground with an HM at 450 µm, RM at 450 µm, or no further ground at 670 µm^*a*^, as-is basis

	HM, 450 µm					RM, 450 µm					Unground, 670 µm				
	Sieve screen opening^*b*^, µm					Sieve screen opening, µm					Sieve screen opening, µm				
Item	212	300	425	600	850	212	300	425	600	850	212	300	425	600	850
DM, %	92.4	92.0	91.9	92.1	92.2	92.9	92.4	92.2	91.5	92.4	92.9	92.2	92.7	92.8	93.0
GE, Mcal/kg	4.65	4.67	4.63	4.64	4.78	4.71	4.73	4.62	4.66	4.67	4.65	4.73	4.72	4.73	4.78
CP, %	30.6	29.0	28.6	28.2	28.0	32.8	31.5	30.6	29.1	24.8	33.0	31.1	29.9	28.8	28.9
AEE, %	7.4	7.6	8.4	9.3	10.1	7.1	8.0	8.5	9.3	7.9	7.4	7.8	7.6	8.8	10.0
NDF, %	27.8	30.0	31.1	29.9	26.4	34.3	25.8	24.8	25.7	24.6	25.6	29.3	29.0	26.4	25.4
Starch, %	12.3	12.4	11.7	12.6	13.5	13.3	12.4	13.0	13.9	12.6	11.3	10.8	11.1	10.8	12.3

^*a*^Analysis was performed in duplicate from the particles retained in each sieve fraction.

^*b*^Sieve screen opening sizes: 212, 300, 425, 600, and 850 µm correspond to the U.S. standard sieve numbers 70, 50, 40, 30 and 20, respectively.

Crude protein concentrations were slightly decreased as sieve opening increased in all the three GMs tested. The concentration of AEE increased as sieve opening increased in unground DDGS and HM, while it seemed to be constant for RM.

## DISCUSSION

At the feed mill, corn DDGS may be ground to a smaller PS; depending on available equipment, an HM or RM may be used. The purpose of such grinding is to increase feeding value by enhancing the digestibility of energy and nutrients (Htoo et al., 2008; Acosta et al., 2020a, 2020b). The results of this experiment confirmed that reducing the PS of corn DDGS with either an HM or an RM (from 670 to 450 μm) increased the digestibility of DM, GE, and AEE and tended to increase the digestibility of N. This improvement is presumably a consequence of increasing the surface area (Stark, 2012), allowing for easier access by enzymes secreted within the gastrointestinal tract of the pig. It may also be due to the release of nutrients resulting from the disruption of the fiber matrix of the feed ingredient (Htoo et al., 2008). As mentioned in the introduction, Liu et al. (2012) observed an increase in the ATTD of DM and GE by reducing PS from 818 to 308 μm using an HM. Similarly, Yáñez et al. (2011) reported increased digestibility of GE and N between two PS (517 and 383 μm) in cofermented corn and wheat DDGS ground with an HM.

However, data comparing GMs for corn DDGS could not be found in the literature. This information is needed since mill types can produce different grinding products. Specifically, differences in the shape of the particles of each GM have been reported (Reece et al., 1985; Wondra et al., 1995; Hancock and Behnke, 2001). Also, there is a marked difference in the PS distribution between RM and HM. In general terms, RM has a narrower distribution than HM (Nir et al., 1995; Xu et al., 2015; Vukmirović et al., 2016). This implies that there are more particles around the desired PS with an RM and a greater spread of PS along with the different size categories with HM. Also, it has been reported that ingredients ground using an RM have a lower percentage of fines than HM (Nir et al., 1990; Svihus et al., 2004). In this experiment, scanning electron microscopy images did not reveal evident differences in the shape of particles between the two GM. Additionally, although the comparison between PS distributions showed a slightly narrower distribution for RM than HM, it was less apparent than those observed for corn by Acosta et al. (2020b). The lack of a marked differences in shape and distribution can be the consequence of corn DDGS being reground. When grains are reground, particles are more homogeneous and may share parts of the shape of the previous grinding (Al-Rabadi, 2018). Additionally, corn DDGS is the result of an exhaustive process that involves several steps, including enzymatic hydrolysis, fermentation, heating, and centrifugation. Definitively, these processes also define the shape and structure of the particles. Regrinding is only an additional step in the particle formation for corn DDGS.

Independent of GM, these results suggest that BW increases digestibility. Mainly, a large improvement was observed for the ATTD of N, a moderate increase for the digestibility of GE, and a modest increase for the digestibility of DM. Similar results were obtained by Acosta et al. (2020b) studying the digestibility of corn. Although not fully studied, this effect has been attributed to the greater development of the gastrointestinal tract as pigs grow (Noblet and Henry, 1993; Noblet and van Milgen, 2004). On the other hand, the ATTD of AEE decreased between growing and finishing pigs. A decrease in fat digestibility as pigs grow is unexpected and difficult to explain on the surface. Looking more closely at fat digestion, this response may very well be related to differences in endogenous intestinal secretions between BW categories (Kellner et al., 2018). In this case, it is possible that greater endogenous losses of AEE in finishing pigs may lower the apparent digestibility of AEE compared with growing pigs. However, since there are no comparable data, it is necessary to investigate this effect further.

Unlike other dietary components, the insoluble fiber fraction of corn DDGS was not affected by reducing PS, suggesting that increasing surface area does not enhance the fermentation of its structural carbohydrates. Furthermore, results also indicate that the digestibility of insoluble fiber from corn DDGS does not improve at heavier BW. Previous literature in corn and wheat (Acosta et al. 2020a, 2020b) suggested that PS and BW influence the digestibility of the insoluble fiber components. However, in the case of corn DDGS, this difference from the whole grain products might be the result of the multiple processes involved in its production. Additionally, the elevated level of insoluble fiber in corn DDGS can be a limiting factor in its utilization since pigs have limited fermentation capacity (Acosta et al., 2020c). Thus, although reducing the PS of corn DDGS is an effective way to increase the digestibility of numerous nutrient fractions, it does not seem to be a viable strategy to enhance the utilization of insoluble fiber in growing pigs.

The chemical composition of sieve fractions (from 212 to 850 μm) was analyzed to assess if there was a separation of components of the grain between small and larger particles. If this was the case, it may result in reduced accessibility to the functioning of endogenous enzymes secreted by the gastrointestinal tract of the pig. On the other hand, if certain nutrients are concentrated at finer PS, they may be rendered more digestible. The results reported herein suggest that the distribution of DM, GE, AEE, NDF, and starch is similar across PS and, therefore, would not be a factor in differential digestion. Crude protein acted differently as it was more concentrated in the smaller sieve fractions, especially for corn DDGS ground with an RM. Liu (2008) evaluated the chemical composition of seven sieve fractions (from 110 to 2,360 µm) of corn DDGS from 11 ethanol plants and reported that fat and CP were more concentrated in the smaller particles. Other authors have also reported CP being segregated among particles in a similar manner (Liu, 2009b; Cheng and Rosentrater, 2017). Overall, these results suggest that corn DDGS maintains much of the chemical composition from the original ingredient across the different PS.

In conclusion, reducing PS of corn DDGS from 670 to 450 μm either with an HM or with an RM improved the digestibility of DM, GE, and AEE and modestly improved the digestibility of N in growing and finishing pigs. However, reducing the PS of corn DDGS did not affect the digestibility of fiber components.
